# Genomic Comparison of Endometrioid Endometrial Carcinoma and Its Precancerous Lesions in Chinese Patients by High-Depth Next Generation Sequencing

**DOI:** 10.3389/fonc.2019.00123

**Published:** 2019-03-04

**Authors:** Yao Wang, Mei Yu, Jia-Xin Yang, Dong-Yan Cao, Ying Zhang, Hui-Mei Zhou, Zhen Yuan, Keng Shen

**Affiliations:** Department of Obstetrics and Gynecology, Peking Union Medical College Hospital, Chinese Academy of Medical Sciences and Peking Union Medical College, Beijing, China

**Keywords:** endometrioid endometrial cancer, endometrial intraepithelial neoplasia, next generation sequencing, predisposing genes, carcinogenesis

## Abstract

Endometrial intraepithelial neoplasia (EIN), also known as endometrial atypical hyperplasia (EAH) is believed to be the precursor lesion of endometrioid endometrial carcinoma (EEC). Many genetic factors play important roles in the process of carcinogenesis, however, the key genetic alterations from dysplasia to endometrial cancer remains poorly understood. Germline mutations in Lynch syndrome genes are associated with hereditary endometrial carcinoma. The role of other cancer susceptibility genes is unclear. The aim of this study was to investigate the genomic alterations of premalignant endometrial lesion and EEC, and to determine the prevalence of cancer predisposition gene mutations in an unselected endometrial carcinoma patient cohort. Here, we applied a comprehensive cancer gene panel (363 cancer-related genes) to capture the exomes of cancer-related genes. Samples were collected from 79 patients with EEC and 36 patients with EIN. Our results demonstrate that EIN harbors most of the driver events reported in EEC and for the first time we reported a high frequency of the amplification of VEGFB gene in endometrial cancer. Moreover, we identified four novel candidate cancer-associated genes (CTCF, ARHGAP35, NF1, and KDR) which may be crucial in the carcinogenesis of EEC. In addition, we identified 2 patients who had a deleterious germline mutation in Lynch syndrome genes (MLH1 and MLH2), and another 8 patients harbored germline mutations of 6 non-Lynch syndrome genes (MUTYH, GALNT12, POLE, MPL, ATM, and ERCC4) which may be associated with endometrial cancer. Larger series will have to be investigated to assess the risks and the proportion of endometrial cancers attributable to other genes.

## Introduction

Endometrial cancer (EC) is the most frequent gynecological cancer in developed countries, globally affecting more than 380,000 new women each year (1). In recent years, with the rapid development of the economy in China, people's living habits and dietary structure have undergone great changes. Accompanied by the increase of metabolic diseases, the incidence of endometrial cancer is gradually increasing. In China, endometrial cancer ranks second among gynecological cancers in its incidence, with approximately 63,400 new cases diagnosed in 2015 (2). Approximately 80% of all endometrial carcinomas are of endometrioid endometrial carcinoma (EEC), which are associated with long stimulation of estrogen without antagonism of progestogen and have a favorable prognosis (3). The genetic landscape of EEC has been characterized in 2013 by TCGA, which reveals frequent mutations in PTEN, CTNNB1, PIK3CA, ARID1A, KRAS, and novel mutations in the SWI/SNF chromatin remodeling complex gene ARID5B (4).

Endometrial intraepithelial neoplasia (EIN) is considered to be the precursor lesion of EEC. Approximately 20% of the EIN will progress into EC, but the molecular pathogenesis underlying this progression is poorly explored. Thus, there is a demand for comprehensive exploration of the underlying genetic ordering events. Previous studies demonstrate that EIN share genetic similarity with EEC such as microsatellite instability(MSI) and somatic mutations of PTEN, PIK3CA, CTNNB1, and KRAS (5–9). In addition, EIN also have notable different specific mutations, which suggests activation of specific pathways by different mutational mechanisms. However, due to the small sample size and limited detected genes, researchers did not find any statistically significant genetic differences between EIN and EEC and the difference of molecular profiles between them still remains obscure (8). Which molecular variations play crucial role in initiating the progression from dysplasia to carcinoma has not been determined. Hence, it is important to explore the molecular features of EIN and EEC, which may also be valuable for the clinical management.

Lynch syndrome (LS) is a hereditary cancer syndrome caused by germline mutations in the mismatch repair (MMR) genes (MLH1, MSH2, MSH6, and PMS2), which has been regarded as the most common hereditary cancer syndrome (10). Patients of LS have an increased lifetime risk of colorectal, endometrial, ovarian, and many other cancers (11). In addition to LS, other hereditary syndromes characterized by deleterious germline mutations in other genes (such as PTEN, MUTYH) are less common and rarely investigated in Chinese population (12).

Although previous studies detailed the patterns of somatic alterations across primary endometrial tumors. To our knowledge, no comparative study has reported on molecular profiles of premalignant endometrial lesions and endometrioid endometrial cancer in Chinese population. Such information would be helpful in understanding the underlying genetic ordering events in endometrial cancer progression. Herein, we performed high-depth targeted sequencing to detect the mutational status in 363 cancer-related genes from 115 paired fresh-frozen tissues and whole blood samples of patients within EIN and EEC.

## Materials and Methods

### Patients and Samples

We obtained fresh-frozen tumor blocks and paired germline blood samples from 115 individuals (36 with EIN and 79 with EEC) who underwent surgery between September 2017 and September 2018 at Peking Union Medical College Hospital (PUMCH). Patients' characteristics are listed in Table 1. The diagnosis of cases was confirmed by at least 2 experienced gynecological pathologists. Tumor staging and grading were performed according to International Federation of Gynecology and Obstetrics (FIGO) 2014 standards. This study was approved by the Ethics Committee of PUMCH, Beijing, China (HS-1704). Written informed consent was obtained from all participants of this study at admission to PUMCH.

**Table 1:** Patient demographics in the high-throughput sequencing experiment.

**Characteristics**	**Classification**	
Age, median (range)	EIN	36 (20–61)
	EC	57 (26–80)
Pathological classification	EIN	36
	G1 EEC	40
	G2 EEC	24
	G3 EEC	15
FIGO staging of EC	IA	61
	IB	4
	II	3
	IIIA	4
	IIIIB	2
	IIIC	4
	IV	1

*EIN, endometrial intraepithelial neoplasia; EEC, endometrioid endometrial carcinoma; EC, endometrial cancer*.

### DNA Isolation, Library Construction, and Amplification, Targeted Capture, and Illumina-Based Sequencing

Genetic testing was performed after pathological diagnosis of EIN and EEC. Genomic DNA was extracted from fresh frozen tissue (somatic) and the matched blood sample (germline) using the DNeasy Blood & Tissue Kit (Qiagen, Hilden, Germany) according to the manufacturer's instructions. DNA was purified by AxyPrep™ Mag Tissue-Blood genomic DNA Kit (Axygen, America). And its quantification was measured by the Qubit dsDNA HS Assay Kit (Life Technologies, Eugene, OR). All purified DNA samples are judged to be of high quality (concentration > 3.4 ng/ul) for mutation analysis. Fifty nanograms of genomic DNA was fragmented randomly into fragments which size range from 200 to 300 bp. Fragmented DNA was added with barcode and adaptors using polymerase chain reaction (PCR) reagents, the quality of PCR products was checked. The products were used for library construction and follow-up exon capture. Captured fragments were subsequently purified, amplified, ligated, and circularized by NimbleGen SeqCap EZ Hybridization and Wash Kit (Roche NimbleGen, Madison, WI). Finally, high-throughput sequencing of library products was performed on NextSeq CN500 (BerryGenomics, China).

### SNV and INDEL Calling

After the sequencing, the FASTQ file was used for alignment and variant calling. To filter poor-quality reads, flexbar V2.4 software was used to process the raw read data files by removing the sequence of the original reads data and low-quality sequenced bases. The retained sequencing reads were then aligned to the reference human genome (NCBI Human Genome Buid37, hg19) using BWA (Burrows-Wheeler Aligner, version 0.5.9) software. SAM tools (Utilities for the Sequence Alignment/Map format, version 1.57) were used to integrate the matching information. GATK (Genome-Analysis Tool Kit, version 3.6), a widely used genetic variants discovery tool, was applied to identify single nucleotide variations (SNVs) and INDELs according to the sequence alignment results. Other databases and tools were also used to annotate identified genetic variants, including Variant Tools Version 3.0 (https://vatlab.github.io/vat-docs/), ANNOVAR Version 3.5a (http://annovar.openbioinformatics.org/en/latest/), dbSNP database (https://www.ncbi.nlm.nih.gov/SNP/), Clinvar (https://www.ncbi.nlm.nih.gov/clinvar/), Cosmic V86 (https://cancer.sanger.ac.uk/cosmic/). Tumor mutational burden (TMB) was determined by the number of SNVs (depth >150X and allele frequency ≥ 0.03) which was detected on NGS (interrogating Mb of the genome), and the value was extrapolated to the whole exome using a validated algorithm.

### Determination of Potential Driver Mutations

A bioinformatic algorithm called MutSigCV (13) was to predict the probability that an individual gene functions as a driver gene in the initiation and progression of endometrial cancer. As for several specific genes not listed in the MutSigCV based database, Tumor Suppressor and Oncogene (TUSON) Explorer (14), a computational method that analyses the patterns of mutational signatures in tumors and predicts the likelihood that any individual gene functions as a tumor suppressor (TSG) or oncogene (OG) was applied.

### Copy Number Analysis

Copy number variation (CNV) was called from read counts by algorithm written by Berry Genomics. The details of this algorithm are as follows: After obtaining the sequencing data alignment information, the gene is divided into multiple regions and the average sequencing depth of each region is counted. Then, the sequencing depth of each region and its corresponding normal tissue sample is compared and the LRR value (LRR = $\log_{\text{2}}\frac{sequencing\;depth\;of\;the\;tumor\;sample}{sequencing\;depth\;of\;the\;normal\;tissue\;sample}$) of each region is calculated. According to LRR value, determine whether each region is amplified (LRR > 0.35) or deleted (LRR < 0.5). Finally, the proportion of the number of amplified and deleted regions of each gene is counted, and the type (amplification or deletion) which accounts for more than 70% is considered as the copy number variation type of the gene. Functional annotation of the differentially mutated genes was performed by use of the Gene Set Enrichment Analysis (GSEA) (http://software.broadinstitute.org/gsea/index.jsp).

### Statistical Analysis

The Pearson's χ2-test or the Fisher exact test was used for a comparison of frequencies between two groups. All tests were two sided, and *P* < 0.05 was considered statistically significant. All statistical analyses were performed with SPSS version 22.0 software (SPSS Inc., Chicago, IL, USA).

## Results

### Overview of Sequencing Data

DNA from all samples was successfully extracted and amplified by multiplex PCR, which was all qualified for library construction and next-generation sequencing. The genomic profiles of EIN and EEC were analyzed by a designed panel which targeted 363 cancer-related genes (Supplementary Figure 1). On average, we obtained 27 million 50-nt single reads for tumor samples and 23 million reads for matched blood samples. For the targeted regions, the average depth of coverage was 1690 × and 2389 × for tumor and matched blood samples, respectively. The average coverage rate of the target region was 99.35% (Supplementary Table 1).

**Figure 1:**
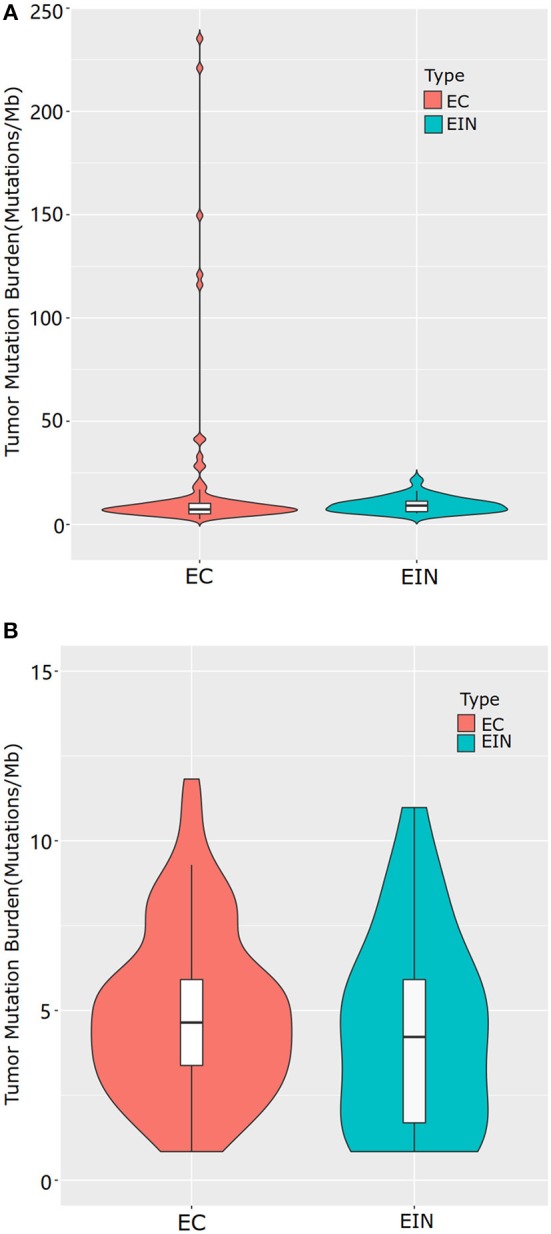
Tumor mutational burden levels of EEC and EIN. **(A)** comparison of TMB which included all samples of EEC. **(B)** comparison of TMB which excluded samples of EEC with mutations in genes involved in DNA damage repair pathway. EIN, endometrial intraepithelial neoplasia; EEC, endometrioid endometrial carcinoma.

Sequencing data for all samples meets somatic mutation analysis requirements. After variant filtration, we identified a total of 1,803 variants in 310 genes, including 1,620 single-nucleotide variants (SNVs) and 183 small scale insertions/deletions (indels) (Supplementary Table 2). All EC cases were identified at least one alteration among the 363 genes screened, while 5 cases of EIN had no somatic mutation identified. Across the entire dataset, the median TMB was 5.07 mutations/Mb, with a range of 0–233.93 mutations/Mb. For all ECCs, the median TMB was 5.49 mutations/Mb, with a range of 0.84–233.93 mutations/Mb. For all EIN, the median TMB was 2.955 mutations/Mb, with a range of 0–16.05 mutations/Mb. Patients with EEC had a significantly higher median TMB than patients with EIN (5.49 vs. 2.955, *p* = 0.000) (Figure 1A).

There were five highly mutated patients (median 173 per tumor, range from 141 to 277) and the remaining 110 patients were non-highly mutated (median 6 mutations per tumor; range 0–48). We further investigated and found that hypermutated phenotype group could be attributed to a disturbed DNA repair system resulted from the mutated genes involving in DNA damage repair pathway such as POLE, POLQ and mismatch repair (MMR) genes (MSH2, MSH6, MLH1, MLH3, PMS1, and PMS2). Details of the mutated genes in DNA damage repair pathway were shown in Supplementary Table 3. To distinguish the actual difference of TMB between EEC and EIN, we excluded samples with mutations in DNA damage repair pathway and the result showed the median TMB of two groups had no significant difference (5.07 vs. 3.80, *p* = 0.294) (Figure 1B).

### Genomic Profiles of Premalignant Endometrial Lesions and Endometrial Cancer

#### Mutated Genes and Predicted Driver Genes

**Figure 2:**
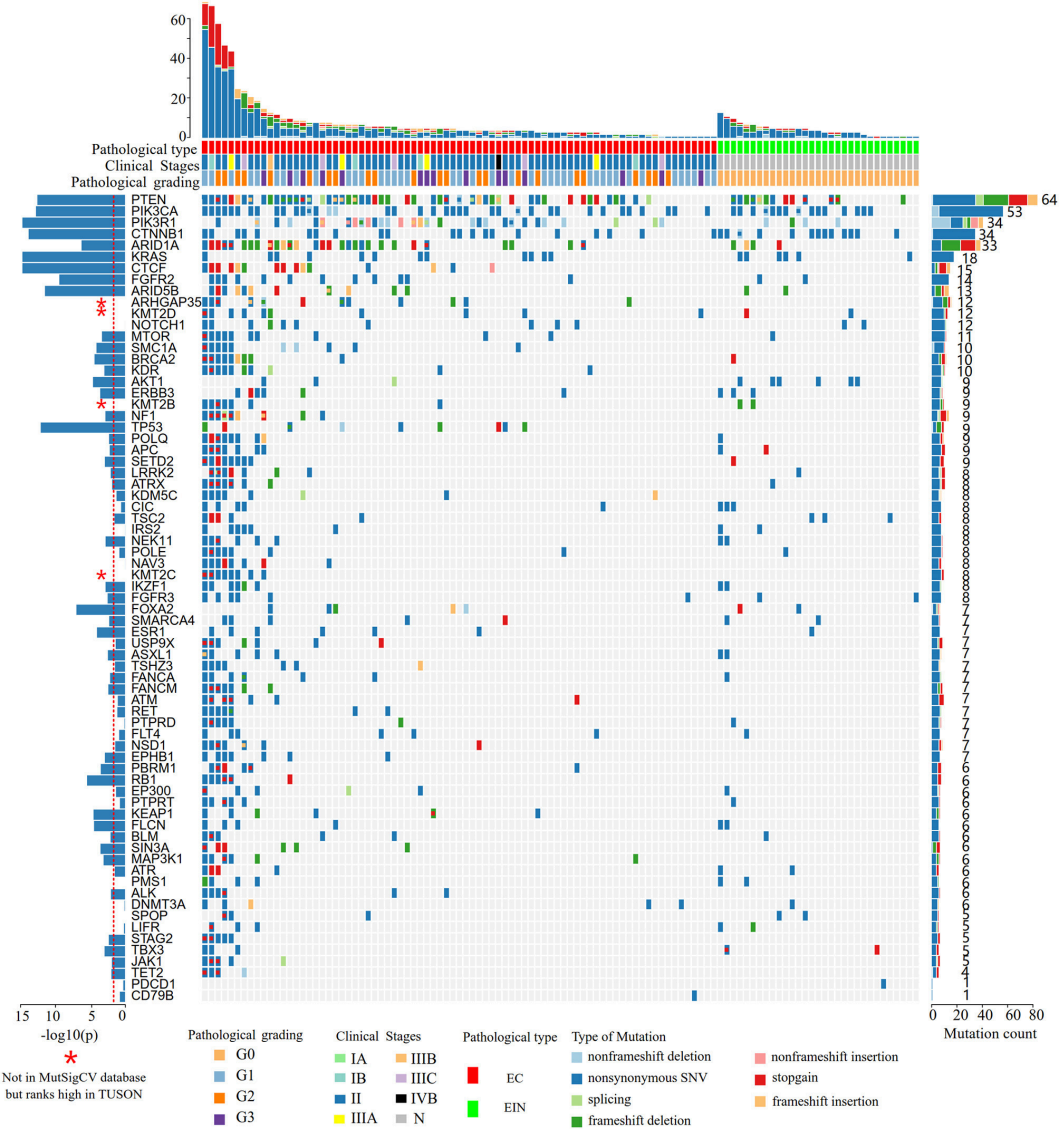
Heat map showing somatic mutation profiles of EIN and EEC. Top, the number of different types of mutations in each sample. Middle, mutated genes are ranked based on the mutation frequency. Left, significance levels (*P*-values) of each gene predicted to be drive gene by MutSigCV. The red dotted line indicates that the *P*-value is 0.01, and the bar above the line indicates that the *P* < 0.01. Right, the frequency of each mutated gene in all samples. EIN, endometrial intraepithelial neoplasia; EEC, endometrioid endometrial carcinoma.

In all cases, the most frequently altered genes were PTEN (53.9%), PIK3CA (46.1%), CTNNB1 (29.6%), PIK3R1 (29.6%), ARID1A (28.7%), KRAS (11.5%), CTCF (13.0%), FGFR2 (12.2%), and ARID5B (11.3%) (Figure 2). PTEN, PIK3R1, and ARID1A alterations were significantly more commonly occurred in cases of endometrial cancer than in EIN (*p* all < 0.05) (Table 2). We find that 4 cases (11.1%) with EIN and 24 cases (30.4%) with EEC have concurrent mutations of PTEN and PIK3CA (*P* = 0.046). Simultaneous mutations of PTEN and PIK3R1 genes were identified in 23 cases, and the occurrence rate in EC is higher than EIN with no significant difference (24.1% vs. 11.1%, *P* = 0.175). CTNNB1 mutations were identified in 29.6% (34/115) samples, with 38 non-synonymous variants and 1 non-frameshift deletion detected. 79.5% (31/39) variants occurred in exon 3. T41I (c.122C > T) is the most frequently recurrent variant followed by S37F (c.110C > T) and R93M (c.278G > T). According to the JAX-Clinical Knowledgebase annotation, 31 variants can lead to an accumulation of CTNNB1 expression products which can activate cell proliferation. In addition, pathological examination revealed that all samples harboring CTNNB1 mutation were well or moderate differentiated. KRAS mutations were identified in 15.7% (18/115) cases, and 88.9% mutations occurred in exon 2. Of three recurrent variants, G12V (c.35G > T) is the most commonly (38.9%) occurred followed by G12D (c.35G > A, 27.8%) and Q61H (c.183A > C, 11.1%). ARID1A mutations were identified in 27.8% (32/115) samples, and a total of 49 variants were detected. 42.9% (21/49) mutations occurred in exon 20. But only R1989X was recorded in present database which was a pathogenic variant.

**Table 2:** The highly mutated genes (mutation frequency ≥ 9) in all samples.

**Gene symbol**	**Total prevalence**	**MutSigCV *P*-value**	**Pathway**	**EEC group**	**EIN group**	***P*-value**
PTEN	64/115 (53.9%)	5.88 × 10^−15^	PI (3)K pathway	51/79	13/36	**0.008**
PIK3CA	53/115 (46.1%)	3.44 × 10^−15^	PI (3)K pathway	37/79	16/36	0.811
CTNNB1	34/115 (29.6%)	2.22 × 10^−16^	RTK/RAS/β-catenin	22/79	12/36	0.607
PIK3R1	34/115 (29.6%)	2.22 × 10^−17^	PI (3)K pathway	30/79	4/36	**0.004**
ARID1A	33/115 (28.7%)	8.78 × 10^−8^	SNF/SWI	29/79	4/36	**0.007**
KRAS	18/115 (15.7%)	2.22 × 10^−17^	RTK/RAS/β-catenin	12/79	6/36	0.840
CTCF	15/115 (12.2%)	2.22 × 10^−17^	–	15/79	0/36	**0.003**
FGFR2	14/115 (12.2%)	2.17 × 10^−11^	RTK/RAS/β-catenin	12/79	2/36	0.247
ARID5B	13/115 (11.3%)	9.96 × 10^−14^	–	11/79	2/36	0.340
NOTCH1	12/115 (10.4%)	0.997	Notch signaling pathway	9/79	3/36	0.866
ARHGAP35	12/115 (10.4%)	1	–	12/79	0/36	**0.009**
KMT2D	12/115 (10.4%)	1	–	10/79	2/36	0.409
MTOR	11/115 (9.6%)	1.79 × 10^−4^	PI (3)K pathway	10/79	1/36	0.184
SMC1A	10/115 (8.7%)	2.31 × 10^−5^	–	9/79	1/36	0.168
BRCA2	10/115 (8.7%)	1.12 × 10^−5^	–	9/79	1/36	0.168
KDR	10/115 (8.7%)	4.44 × 10^−4^	–	10/79	0/36	**0.030**
AKT1	9/115 (7.8%)	6.37 × 10^−6^	PI (3)K pathway	3/79	6/36	**0.045**
APC	9/115 (7.8%)	3.28 × 10^−3^	–	8/79	1/36	0.324
KMT2B	9/115 (7.8%)	1	–	7/79	2/36	0.812
ERBB3	9/115 (7.8%)	9.30 × 10^−5^	–	7/79	2/36	0.812
NF1	9/115 (7.8%)	6.56 × 10^−4^	–	9/79	0/36	0.055
TP53	9/115 (7.8%)	2.07 × 10^−14^	P53 pathway	9/79	0/36	0.055
POLQ	9/115 (7.8%)	2.58 × 10^−3^	–	8/79	1/36	0.324

*Statistically significant values are shown in bold*.

According to the MutSigCV analysis result, 44 genes were predicted to be genetic drivers of endometrial cancer (*P* < 0.01, Figure 2). And the top 9 highly mutated genes (PTEN, PIK3CA, PIK3R1, CTNNB1, ARID1A, KRAS, CTCF, FGFR2, ARID5B) together with TP53 and FOXA2 had stronger tendency to be tumor driver genes compared to other genes (*P* < 10^−7^, Figure 2). To be noticed that, 4 genes (ARHGAP35, KMT2D, KMT2B, KMT2C) were not included in the MutSigCV database, but ranks high in TUSON Explorer (top 100 TSGs), which was shown in Figure 2 and Supplementary Table 2.

#### Copy Number Alteration Data

**Figure 3:**
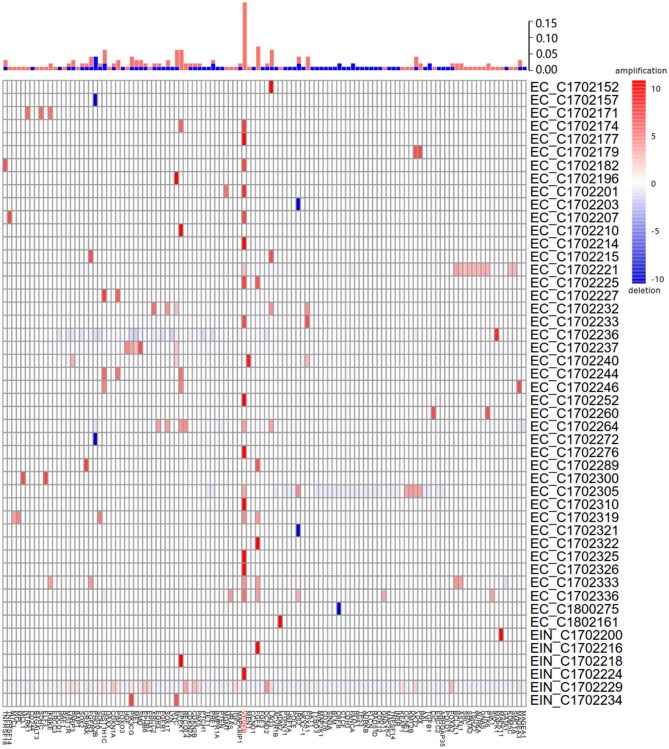
Summary of all copy number aberrations in 48 samples. Upper bar, frequency of amplifications and deletions. Middle bar, heatmap of copy number aberration profiles of 48 samples. Amplified and deleted genomic regions are shown in red and blue, respectively. The high frequency of amplification of VEGFB is easily noticeable.

Of 115 samples, copy number alterations (CNAs) occurred in 48 (41.7%) samples (Figure 3). The overall CNA burden was relatively low (median 2 CNAs per sample, range 0–37). The overwhelming majority of CNAs were amplification of genes, and the amplification of VEGFB gene was the most frequent CNA which was presented in 20/115 samples of which 19/20 were endometrial cancer (95%) and 1/20 were EIN (5%). Indeed, earlier reports reported this gene was associated with tumorigenesis (15).

#### Differentially Mutated Genes

Of the frequently mutated genes, the mutation frequencies of 7 genes (PTEN, PIK3R1, ARID1A, KRAS, CTCF, ARHGAP35, and KDR) had significant difference between EIN and EEC groups (*P* all < 0.05). Besides, mutations of CTCF, ARHGAP35, NF1, KDR, and TP53 were only detected in EEC cases (Table 2; Figure 2). Of these genes, we identified 15 somatic CTCF mutations in EEC samples, and the majority mutations resulted from frameshift (33.3%) and non-sense (46.7%) mutations. And a mutational hotspot p.T204fs was identified in 4 patients. As for mutations of ARHGAP35, 50% mutations resulted from missense mutations, 33.3% resulted from frameshift mutations and the remaining (20%) were non-sense and non-frameshift deletion mutations. Nine patients with EEC harbored NF1 mutations, of which there were two recurrent mutational spots (p.R2450 and p.L2639I). The TP53 mutation frequency in our study was 7.8% and 2 mutations occurred in patients with well-differentiated EEC. A recurrent mutational spot (p.A379V) in KDR gene were identified in 2 patients.

Moreover, we identified the pathways which top 10 differentially mutated genes clustered in (Table 3). The key mutated genes of GO enrichment analysis were Intracellular signal transduction, Positive regulation of biosynthetic process, Regulation of organelle organization and Regulation of cell matrix adhesion. As shown in Table 3, the most significantly mutated genes enriched KEGG pathways were Endometrial cancer, Focal adhesion, Glioma, Melanoma, Small cell lung cancer and Prostate cancer.

**Table 3:** Top 10 significantly mutated GO terms and KEGG pathways.

**Term**	**Description**	**Mutated genes**	***P*-value**	**FDR**
**GO TERM**				
GO:1902531	Intracellular signal transduction	PIK3R1, PTEN, AKT1, TP53, KDR, NF1, ARHGAP35	7.80 × 10^−11^	3.60 × 10^−7^
GO:0009889	Positive regulation of biosynthetic process	PIK3R1, PTEN, AKT1, TP53, KDR, NF1, CTCF, ARID1A	2.34 × 10^−10^	4.36 × 10^−7^
GO:0033043	Regulation of organelle organization	PIK3R1, PTEN, AKT1, TP53, KDR, CTCF	8.01 × 10^−10^	6.17 × 10^−7^
GO:0007160	Regulation of cell matrix adhesion	PIK3R1, PTEN, KDR, NF1	2.86 × 10^−9^	1.32 × 10^−6^
**KEGG PATHWAY**				
hsa05213	Endometrial cancer	PIK3R1, PTEN, AKT1, TP53	3.04 × 10^−10^	4.36 × 10^−7^
hsa04510	Focal adhesion	PIK3R1, PTEN, AKT1, KDR, ARHGAP35	3.77 × 10^−10^	4.36 × 10^−7^
hsa05214	Glioma	PIK3R1, PTEN, AKT1, TP53	7.60 × 10^−10^	6.17 × 10^−7^
hsa05218	Melanoma	PIK3R1, PTEN, AKT1, TP53	1.09 × 10^−9^	7.20 × 10^−7^
hsa05222	Small cell lung cancer	PIK3R1, PTEN, AKT1, TP53	2.16 × 10^−9^	1.25 × 10^−6^
hsa05215	Prostate cancer	PIK3R1, PTEN, AKT1, TP53	2.86 × 10^−9^	1.32 × 10^−6^

*FDR, false discovery rate*.

#### Germline Mutations

Table 4 summarizes age at diagnosis, tumor histology, and family history for patients with deleterious germline mutation in Lynch and non-Lynch syndrome genes. There were no germline mutations detected in EIN, whereas 10 patients with EEC (10/115) harbored 10 pathogenic or likely pathogenic germline mutations in 8 different genes. Notably, identification of a germline MMR mutation was detected in 2 patients with EEC (2/79, 2.5%). And both of them had also undergone immunohistochemistry expression of mismatch repair proteins for evaluation of possible Lynch Syndrome after surgery. On patient had loss of MLH1 and PMS2 expression, and the other had loss of MSH2 and MSH6 expression. Eight patients were identified with germline mutations in non-Lynch syndrome genes. Two endometrial cancer patients were identified with MUTYH homozygous c.934-2A>G or heterozygous c.G467A (p.W156X) mutation. And one patient harbored a pathogenic variant of MPL (c.981-1G>C). Potentially pathogenic variants were found in five Cancer Gene Census germline genes: GALNT12, POLE, MPL, ATM, and ERCC4.

**Table 4:** Ten patients with germline mutations in 363 genes detected using NGS of 115 paired samples.

**Gene**	**Base change/Variant effect**	**Mutant type**	**ClinVar/COSMIC**	**Age at diagnosis**	**Histology**	**Family history**
MUTYH	c.934-2A>G	hom	Pathogenic	34	G1EEC	None
MLH1	c.885-1_893delGTTTAGAAAT	hom	Pathogenic	28	G3 EEC	CRC-FDR, SDR
MSH2	c.C1183T (p.Q395X)	hom	Pathogenic	49	G1 EEC	None
GALNT12	c.G5A (p.W2X)	hom	Likely pathogenic	26	G1 EEC	None
POLE	c.4430delG (p.S1477Tfs)	het	Likely pathogenic	63	G1 EEC	CRC-FDR
MPL	c.313_316delTTTC (p.F105Rfs)	het	Likely pathogenic	54	G1 EEC	None
ATM	c.C8494T (p.R2832C)	het	Likely pathogenic	61	G2 EEC	None
MUTYH	c.G467A (p.W156X)	het	Pathogenic	51	G2 EEC	None
ERCC4	c.1536dupA (p.G513Rfs)	het	Likely pathogenic	57	G3 EEC	None
MPL	c.981-1G>C	het	Pathogenic	45	G1 EEC	None

*CRC, colorectal cancer; FDR, first-degree relative; SDR, second-degree relative; het, heterozygous; hom, homozygous; EEC, endometrioid endometrial carcinoma*.

## Discussion

Regarding molecular evolution of EIN and EEC, EIN is the result of a series of mutations involving multiple genes, including tumor suppressor genes, oncogenes, mismatch repair, and genes that control cell growth, differentiation and apoptosis (16). Not all precursor lesions of endometrial cancer can progress to carcinoma, therefore, it is of great importance to distinguish which patients with EIN will develop to EC, and this will be helpful for clinicians to tailor therapeutic interventions. Previous studies have demonstrated that dysplasia and endometrial cancer share several aberrant characteristics with each other. However, the ordering events from dysplasia to carcinoma and which molecular alteration in precursor lesions can predict cancer progression remain unclear. Recently, Russo et al. performed next-generation sequencing on paired EIN and EEC from a series of hysterectomy specimens, and their result suggested the progression from EIN to EEC was not a linear accumulation of mutational events. However, based on a limited sample size, no difference was observed in mutational burden, CNA burden, or specific mutational genes between EEC and EIN (8). In this study, we screened for mutations in 363 genes in endometrial cancer and its precursor lesions using high-throughput sequencing. We believe that this study will shed new light on fundamental aspects for understanding the molecular pathogenesis of endometrial cancer and may aid in the development of new targeted therapies.

### Major Signaling Pathway

Alterations in PI3K/AKT/mTOR signaling pathway are very common in endometrial carcinoma and its precursor lesions (17, 18). Of the genes involved in this pathway examined in our study, PTEN, PIK3CA, and PIK3R1 all mutated frequently, which was similar to the TCGA study on endometrial carcinoma (4). In an early study with small sample size, researchers explored the status of the PIK3CA gene and its association with PTEN mutations in EIN and EC and found a significantly lower frequency of PIK3CA mutations in EIN (5). While in our study, the frequency of PIK3CA mutations between EIN and EEC is comparable (52.7% vs. 39.4%, *P* = 0.811). However, a significantly lower frequency of PTEN and PIK3R1 mutations in EIN were identified. As a transforming oncogene, mutation of PIK3CA gene is an alternative to PTEN mutation, which also leads to an increase in PI3K/AKT/mTOR pathway activity and are important for the invasive potential (19). Previous studies have showed frequent coexistence of PIK3CA and PTEN mutations (17). In our study, 24.3% (28/115) patients have simultaneous mutations in these two genes, and 85.7% (24/28) simultaneous mutations occurred in EC. These results all suggested PIK3CA mutations may have a synergic effect with PTEN inactivation in the development of endometrial tumors. As for the associated survival outcome, the literature suggests that mutations of PI3K pathway may be associated with worse clinical outcomes (20–22).

WNT/β-catenin signaling pathway is the second most frequently activated pathway in EEC (4), which plays a pivotal role in numerous cellular processes such as cell proliferation, differentiation, and maintenance of pluripotency (23). Mutated Wnt pathway components are causative to multiple growth-related pathologies and to cancer (24). β-catenin which is encoded by CTNNB1 gene, is the key downstream effector of this signaling pathway (25). CTNNB1 mutations have been detected in endometrial hyperplasia, suggesting that these mutations occur in the early stages of the neoplastic process. Researchers have found that CTNNB1 mutations can identify a subset of low grade, early stage endometrial cancer patients (26), which was also proved by our study. Mutation in exon 3 of CTNNB1 gene is classically associated with translocation of the β-catenin protein from the membrane to the nucleus and activation of Wnt/β-catenin signaling (27, 28). Jeong et al. (29) found exon 3 deletion of the CTNNB1 gene in a murine model led to upregulation of the Wnt/beta-catenin pathway and the development of endometrial hyperplasia. In present study, 79.5% mutations of CTNNB1 occurred in exon 3, and according to the gene annotation database, most variants lead to an accumulation of CTNNB1 expression products (β-catenin) which can activate cell proliferation. These features make β-catenin (more specifically, a change in its overall expression and/or subcellular localization) an attractive potential marker for EIN and EEC.

The RAS-RAF-MEK-ERK pathway is frequently mutated in human cancers. Of three RAS isoforms, KRAS is the most frequently mutated in human cancers (30), and KRAS activation has been proved as an early oncogenic event in endometrial carcinogenesis (31), which correlates with mucinous differentiation in cancers (32). Although no mucinous component has been detected in our samples, we still identified 15.7% patients had KRAS mutations, and nearly all the mutations occur in codon 12 or 13 of exon 2 of the gene. The hotspot mutations can lead to a mutant oncoprotein, which can decrease its GTPase activity and increase activation of downstream signaling, stimulating neoplastic transformation (33).

In addition to the above mentioned several pivotal signaling pathways involved in transforming endometrial cells to the primary carcinogenesis and metastasis (34), our study for the first time demonstrated a high frequency of the amplification of VEGFB gene. VEGFB is a member of VEGF family, which regulates the formation of blood vessels and involves in endothelial cell physiology (35). It promotes cancer metastasis through the remodeling of tumor microvasculature, and previous study showed high expression levels of VEGF-B in patients with lung squamous cell carcinoma and melanoma correlated with poor survival (15). Targeting VEGFB may be an important therapeutic approach for cancer metastasis. So far, there is no research on the effect of VEGFB on the prognosis of endometrial cancer, and future studies are required to elucidate it. Moreover, we identified the pathways differentially mutated genes between EEC and EIN involved in, such as intracellular signal transduction, positive regulation of biosynthetic process, regulation of organelle organization and regulation of cell matrix adhesion pathways. These pathways may be involved in the multistep development of endometrial cancer. In the future, clinical trials of drugs for endometrial cancer that target these pathways may be carried out.

### Novel Candidate Genes in Malignant Transformation

One of the major findings of our study is that we found different genetic features between EEC and its precursor lesions. Of highly mutated genes detected, ARHGAP35, CTCF, NF1, KDR, and TP53 mutations were only detected in EC cases. Except TP53, other genes have rarely been explored in endometrial cancer before. ARHGAP35 (also known as p190RHOGAP) encoding glucocorticoid receptor DNA-binding factor-1 is a cancer associated gene with a mutation spectrum suggestive of a tumor suppressor function (36, 37). Activation of ARHGAP35 causes RhoA inactivation and inhibits cell invasion (38), while its inactivation could play a role in tumor development (39, 40). Deletion of chromosomal region encompassing ARHGAP gene has been described in solid tumors (41). Abnormal expression of ARHGAP35 in colorectal cancer patients was associated with poor survival (42). However, inactivating mutation status of ARHGAP35 remains unknown in endometrial carcinoma. Our study for the first time reported that ARHGAP 35 gene mutation in endometrial cancer is a distinguishable mutation characterization from its precursor lesions. According to our MutSigCV result, ARHGAP35 is not predicted to be a true cancer driver gene, but a pan-cancer study suggested it may contribute to oncogenesis of endometrial cancer (43). Future research will investigate its expression and clinical significance in endometrial cancer.

CTCF encoding a highly conserved 11-zinc finger DNA binding protein is mutated in about 15% of endometrial cancer (4). Previous study suggested that CTCF is a tumor suppressor and it can regulate the expression of various cancer-related genes (44–46). Loss of CTCF binding can induce epigenetic silencing of tumor suppressor loci or lead to activation of oncogenes. Marshall et al. (47) identified the pro-tumorigenic roles of CTCF mutations in endometrial cancer, and also showed that CTCF haploinsufficiency was associated with poor prognosis. In this study, we only identified CTCF mutations in EEC, and confirmed a recurrent frameshift defect (p.T204Nfs).

The NF1 gene encodes a protein called neurofibromin that is known to function as a tumor suppressor (48). Currently, a genome-wide association study(GWAS) conducted by O'Mara and Glubb (49) identified 9 new susceptibility loci for endometrial cancer, and one loci is NF1 (17q11.2) which encodes a negative regulator of RAS-mediated signal transduction (48).KDR also known as VEGFR, encodes a receptor of vascular endothelial growth factor and has a pivotal role in promoting cancer angiogenesis (50). Research showed its mutation was associated with pancreatic cancer prognosis (51). However, mutations of this gene have rarely been explored in endometrial cancer. According to our MutSigCV result, it was also a putative cancer-associated driver gene.

Based on the above findings, we surmise that these newly discovered four genes (CTCF, ARHGAP35, NF1, and KDR) may correlate with malignant transformation.

### Germline Mutations

Recently, advanced in the next-generation sequencing technology has begun to reshape the field of cancer genetics. Genetic testing not only helps in the diagnosis of cancer and the selection of targeted drugs, but the identification of causative genetic mutations helps predict cancer risk and even achieve cancer prevention through intensive screening and surgical prophylaxis (52). More than 100 cancer predisposition genes have been identified to date (53, 54), while the relationship between susceptibility gene and relevant tumor types still needs further investigation. Most recently, a study which included 10,389 cases across 33 different cancers identified pathogenic or likely pathogenic variants in 8% of all patients (55). However, the genetic architecture of endometrial cancer susceptibility is not well-known. Knowledge of genetic susceptibility to endometrial cancer mainly comes from several hereditary cancer-predisposing syndromes.

Lynch syndrome (LS) is described as an inherited predisposition to colorectal cancer and other cancers, including EC and OC (56). It is caused by autosomal dominant mutations in DNA mismatch repair genes (MLH1, MSH2, MSH6, and PMS2), and women with Lynch syndrome have a cumulative lifetime risk of endometrial cancer of 20–70% (57). In our cohort, 8.7% of patients were found to have pathogenic or likely pathogenic germline mutations. Two of them were carriers of deleterious mutation of MMR genes. One of the patients had a family history of cancers that met the Amsterdam criteria II. The other patient had no family history of any cancer, and had a concurrent stage IA ovarian endometrioid adenocarcinoma.

In our study, 8 patients were identified with pathogenic or likely pathogenic germline mutations in non-Lynch syndrome genes (MUTYH, GALNT 12, POLE, MPL, ATM, and ERCC4), which indicated that there may be other genes outside of Lynch syndrome associated with endometrial cancer. In the future, a germline multi-gene panel targeted on endometrial cancer may be applied to identify additional actionable mutations in endometrial cancer. Germline biallelic inactivation of MUTYH represents a familial cancer syndrome, and patients of bi-allelic MUTYH mutation carriers have an increased risk of developing colorectal cancer (58, 59). At present there is little information about the role of MUTYH in other types of cancer. Biallelic MUTYH mutations have been found in patients affected with endometrial carcinoma, and a few reports indicated a possible relationship with endometrial cancer (60). In our cohort, we identified 2 patients carried pathogenic mutations of MUTYH gene, and only one was bi-allelic which was considered deleterious.

In addition to MUTYH, there were another five genes which were identified with germline variants in patients with endometrial cancer. However, there are no reports on the relationship between MPL, GALNT12 and endometrial cancer to date. Interestingly, the other three germline mutated genes (ATM, ERCC4, and POLE) are all involved in the DNA damage repair pathway (61), which are key factors in maintain genomic integrity and stability. Germline mutations in ATM gene are thought to contribute to breast cancer susceptibility (62). And previous study suggested germline mutations affecting the exonuclease domain of POLE predispose to CRC and endometrial cancer (63). Whether these additional gene mutation carriers are susceptible to endometrial cancer should be explored in future studies.

## Conclusion

Our study, for the first time, portrayed the mutational spectra of both EEC and its precursor lesions in Chinese population. According to our data, EC bears a higher mutational burden than its precursor lesion which attribute to a disturbed DNA repair system resulting from the mutated genes in DNA damage repair pathway. EIN harbored most of the significantly mutated genes which were also prevalent in EEC. We identified several cancer driver genes and defined the pathway involved in the oncogenesis of endometrial cancer. We also identified four novel candidate genes (CTCF, ARHGAP35, NF1, and KDR) which may correlate with malignant transformation of dysplasia. Our findings in germline mutations also suggest that except for Lynch syndrome genes, there are other non-lynch syndrome genes which may associate with endometrial cancer. Larger series will have to be investigated to assess the risks and the proportion of endometrial cancers attributable to other genes. In conclusion, our work represents the beginning step of investigating the genetic relationship between EEC and its precursor lesions. In the future, integrated multi-omics analysis will shed further light on the development of EEC.

## Dataset with Accession Number

The raw sequence data reported in this paper have been deposited in the Genome Sequence Archive (Genomics, Proteomics & Bioinformatics 2017) in BIG Data Center (Nucleic Acids Res 2018), Beijing Institute of Genomics (BIG), Chinese Academy of Sciences, under accession number HRA000033. URL: http://bigd.big.ac.cn/gsa-human/s/U6AiGcb4.

## Author Contributions

MY and J-XY conceived and designed the research. YW and J-XY performed the research. YW is responsible for data analysis and drafted the manuscript. MY and J-XY discussed the results and amended the manuscript. D-YC, YZ, and H-MZ supervised the research. ZY and KS provisioned useful suggestions in table and figure preparation. All authors read and approved the final version of the manuscript.

### Conflict of Interest Statement

The authors declare that the research was conducted in the absence of any commercial or financial relationships that could be construed as a potential conflict of interest.
