# Supplementary material for: Genomic Comparison of Endometrioid Endometrial Carcinoma and Its Precancerous Lesions in Chinese Patients by High-Depth Next Generation Sequencing
Source: Front Oncol. 2019 Mar 4;9:123. doi: 10.3389/fonc.2019.00123 (PMC6410638; doi:10.3389/fonc.2019.00123)
Supplement: Supplementary file 1 [file Data_Sheet_1.pdf]

Supplementary Data Sheet: 363 gene panel list

ABL1	CRIPAK	HGF	MYCN	RPA	B4GALT3
ACVR1B	CRKL	HIST1H1C	MYD88	RPL22	BAGE1
ACVR2A	CSF1R	HIST1H2BD	NAV3	RPL5	BAP1
AJUBA	CTAG2	HNF1A	NBN	RUNX1	BARD1
AKT1	CTCF	HRAS	NCOA4	SDHA	BCL2
AKT2	CTLA4	ICOS	NCOR1	SDHB	BCL2L1
AKT3	CTNNA1	ICOSLG	NEK11	SDHC	BCL6
ALK	CTNNB1	IDH1	NF1	SDHD	BLM
AMER1	DAXX	IDH2	NF2	SETBP1	BMPR1A
APC	DDR2	IGF1R	NFE2L2	SETD2	BRAF
AR	DICER1	IGF2	NFE2L3	SF3B1	BRCA1
ARHGAP35	DNMT3A	IKBKE	NKX2-1	SH2D1A	BRCA2
ARHGEF12	EGFR	IKZF1	NOTCH1	SIK1	BRIP1
ARID1A	EGR3	IL7R	NOTCH2	SIN3A	BTK
ARID2	EIF4A2	INSR	NOTCH4	SLAMF7	BTLA
ARID5B	ELF3	IRF4	NPM1	SMAD2	CARD11
ASXL1	EOMES	IRS2	NRAS	SMAD4	CBFB
ATM	EP300	ITK	NSD1	SMARCA4	CBL
ATR	EPCAM	JAK1	NT5E	SMARCB1	CCND1
ATRX	EPHA1	JAK2	NTRK1	SMC1A	CCND2
AURKA	EPHA2	JAK3	NTRK2	SMC3	CCNE1
AURKB	EPHA3	JUN	NTRK3	SMO	CD160
AXIN2	EPHB1	KDM5C	PALB2	SOCS1	CD244
AXL	EPHB6	KDM6A	PARP1	SOX17	CD274
B2M	EPPK1	KDR	PARP2	SOX2	CD276
ERBB2	KEAP1	PARP3	SOX9	CD28	FLT3
ERBB3	KIT	PAX5	SPATA2	CD38	FLT4
ERBB4	KITLG	PBRM1	SPOP	CD48	FOXA1
ERCC2	KMT2B	PCBP1	SRC	CD69	FOXA2
ERCC3	KMT2C	PCNA	STAG2	CD79B	FOXL2
ERCC4	KMT2D	PDCD1	STK11	CD80	FOXO3
ERCC5	KRAS	PDCD1LG2	SUFU	CD86	FOXP1

ERG	LAG3	PDGFRA	SYK	CDC73	FRK
ESR1	LCK	PDGFRB	TAF1	CDH1	GAGE1
ETV1	LEF1	PHF6	TAS2R38	CDK12	GALNT12
EZH2	LIFR	PHOX2B	TBL1XR1	CDK4	GATA1
FANCA	LIMK1	PIGF	TBX3	CDK6	GATA2
FANCC	LKB1	PIK3CA	TEK	CDK8	GATA3
FANCM	LRRK2	PIK3CB	TET1	CDKN1A	GNA11
FBXW7	LYN	PIK3CD	TET2	CDKN1B	GNAQ
FGF19	MAGEA1	PIK3CG	TGFB1	CDKN2A	GNAS
FGF3	MAGEA12	PIK3R1	TGFBR2	CDKN2B	GREM1
FGF4	MAGEA3	PIK3R2	TLR4	CDKN2C	H3F3C
FGFR1	MAGEA4	PIM1	TNF	CEBPA	HAVCR2
FGFR2	MAGEC2	PMS1	TNFAIP3	CHEK1	HDAC1
FGFR3	MAP2K1	PMS2	TNFRSF14	CHEK2	HDAC2
FGFR4	MAP2K2	POLD1	TNFRSF18	CIC	HDAC3
FH	MAP2K4	POLE	TNFRSF4	CREBBP	HDAC6
FLCN	MAP3K1	POLQ	TNFSF11	TNFSF18	TOP1
FLT1	MAPK1	PPM1D	TNFSF14	TNFSF4	TP53
MAPK11	PPP2R1A	TSHZ3	MPL	RAD50	MLH3
MAPK8IP1	PRDM1	TYRO3	MRE11	RAD51C	RAD21

MCL1	PRF1	U2AF1	MSH2	RAD51D	REL
MDM2	PRKAR1A	USP9X	MSH3	RAF1	MLH1
MDM4	PRX	VEGFA	MSH6	RARA	PTPRT
MECOM	PTCH1	VEGFB	MST1R	RB1	XRCC2
MEN1	PTEN	VEZF1	MTOR	RECQL	TSC2
MERTK	PTK6	VHL	MUTYH	RECQL4	TSHZ2
MET	PTPN11	VTCN1	MYC	RET	
MGMT	PTPRD	WT1	MYCL1	ROS1	
MITF	PTPRK	XPO1	TSC1	TSHR	
