# Supplementary material for: Genomic Comparison of Endometrioid Endometrial Carcinoma and Its Precancerous Lesions in Chinese Patients by High-Depth Next Generation Sequencing
Source: Front Oncol. 2019 Mar 4;9:123. doi: 10.3389/fonc.2019.00123 (PMC6410638; doi:10.3389/fonc.2019.00123)
Supplement: Supplementary file 4 [file Table_3.docx]

| Patient sample ID | Mutated genes in DNA repair pathway | | TMB (mutations/Mb) |
| --- | --- | --- | --- |
|  | Gene | Mutation |  |
| C1702182-T | MSH2  MSH6  POLE  POLQ | c.2101G>T(p.E701X)  c.1445G>A(p.R482Q)  c.2449G>A(p.D817N)  c.890C>T(p.S297F)  c.2997G>T(p.E999D)  c.5863G>A(p.E1955K) | 119.07 |
| C1702185-T | MLH1  MSH3  MSH6  POLE  POLQ | c.1525C>A(p.L509I)  c.4103T>G(p.F1368C)  c.741G>T(p.K247N)  c.1616G>T(p.G539V)  c.261G>T(p.K87N)  c.774C>A(p.F258L)  c.1082G>A(p.R361H)  c.2419G>A(p.E807K)  c.1480C>T(p.R494W)  c.857C>G(p.P286R)  c.2003G>A(p.R668Q)  c.7216G>T(p.E2406X) | 147.79 |
| C1702310-T | MLH3  POLE  POLQ | c.4125G>T(p.Q1375H)  c.1331T>A(p.M444K)  c.3703C>A(p.L1235I)  c.2579G>A(p.R860Q) | 114.01 |
| C1702320-T | MLH3  MSH2  MSH6  PMS1  PMS2  POLE  POLQ | c.3759dupA(p.L1254Ifs)  c.1588G>T(p.E530X)  c.1738G>T(p.E580X)  c.1949G>A(p.G650D)  c.3163G>A(p.A1055T)  c.1442delA(p.N481Mfs)  c.631C>T(p.R211X)  c.1231G>T(p.V411L)  c.6556C>A(p.L2186I)  c.248A>G(p.K83R)  c.2570G>A(p.R857H) | 233.93 |
| C1702223-T | MLH3  MSH2  MSH6  POLE  POLQ | c.1993G>T(p.E665X)  c.199G>T(p.E67X)  c.2684G>A(p.R895H)  c.3833A>G(p.Y1278C)  c.925A>G(p.N309D)  c.1591A>C(p.K531Q)  c.383G>A(p.R128H)  c.1366G>C(p.A456P)  c.5789C>A(p.S1930X)  c.5545C>T(p.R1849X) | 219.57 |

**Supplementary Table 3: Details of mutated genes in DNA repair pathway in 5 highly mutated patients**.
